# Anxiety in women - a Swedish national three-generational cohort study

**DOI:** 10.1186/s12888-018-1712-0

**Published:** 2018-06-04

**Authors:** Gunilla Sydsjö, Sara Agnafors, Marie Bladh, Ann Josefsson

**Affiliations:** 1 Department of Obstetrics and Gynaecology and Department of Clinical and Experimental Medicine, Linköping University, SE-581 85 Linköping, Sweden; 2 Department of Child and Adolescent Psychiatry and Department of Clinical and Experimental Medicine, Linköping University, Linköping, Sweden

**Keywords:** Anxiety, Multi-generation, Women, Offspring

## Abstract

**Background:**

Findings from animal and human studies indicate that anxiety and stress have a negative influence on the child and mother. The aim of this study was to explore the risk for having an anxiety diagnosis and the impact of the diagnosis in a three generational perspective.

**Methods:**

The information was retrieved from Swedish population-based registries. All women who gave birth between 1973 and 1977 (n 169,782), their daughters (n 244,152), and subsequently also the offspring of the daughters (n 381,953) were followed until 2013.

**Results:**

We found that 4% of the mothers and 6% of the grandmothers had been diagnosed with anxiety. Women who had mothers with an anxiety disorder were more than twice as likely to have an anxiety disorder themselves compared to all other women (OR = 2.20, 95% CI = 2.04–2.30). In the third generation, the children born to mothers with an anxiety disorder, the odds ratio of being diagnosed with anxiety was more than twice as high than for the rest of the population (OR = 2.54, 95% CI = 2.01–3.20). If both the mother and the grandmother had had an anxiety disorder the odds ratio for the child having a diagnosis of anxiety was three times higher (OR = 3.11, 95% CI = 2.04–4.75). Anxiety diagnosis in the two previous generations also increased the likelihood of the child having either more than two inpatient visits or more than 10 outpatient visits (OR = 2.64, 95% CI = 2.40–2.91 and OR = 2.21, 95% CI = 2.01–2.43, respectively).

**Conclusions:**

The intergenerational effect on anxiety is high. In order to minimize the risk for further transmission of anxiety disorders, increased awareness and generous use of effective treatment regimes might be of importance.

## Background

Anxiety related diagnoses are prevalent among women in reproductive age groups [1]. In the clinical setting these conditions may be difficult to detect and therefore may be difficult to diagnose. Consequently, women suffering from these conditions are often undertreated or not treated at all. This may lead to a lower quality of life for these women, may have negative effects on family relations, and also have adverse effects on the children’s mental health and wellbeing. Anxiety may also have an impact on the woman’s behavior leading to, for example, problems such as increased drug abuse or becoming prone to accidents, which have a deleterious effect on the woman [2]. It has also been suggested that some individuals with anxiety disorders are genetically predisposed to develop drug addiction [3]. Comorbidity with other mental disorders is common, and the prevalence of individuals with both general anxiety disorders (GAD) and depression is high. In a recent Swedish prevalence study with 3000 participants it was found that among men and women with clinically significant depression or anxiety, nearly 50% had comorbid disorders. The point prevalence of major depression was 5.2, and 8.8% had GAD [4]. Women’s lifetime prevalence of being diagnosed with an anxiety disorder is around 30% [1]. For women of reproductive age, pregnancy and childbirth are sometimes triggers for worsening an already manifested anxiety disorder or for deterioration of an existing anxiety disorder. Thus, pregnant women with mental health problems are common in the antenatal care setting [5, 6]. Anxiety symptoms in pregnant women who may or may not have been given an anxiety diagnosis present themselves in a number of different ways. For example, some of these women might have a severe fear of childbirth, be anxious about the child’s health, anxious about the future, about their relationship with their partner, and even about their new role as a parent [7, 8]. For pregnant women in a population-based community sample, the prevalence of anxiety symptoms was found to be almost 16% in early pregnancy [9]. The study found that women under 25 years of age were at an increased risk for anxiety symptoms during early pregnancy and also revealed that women who were more psychosocially disadvantaged were more often nicotine users before pregnancy. A psychiatric history of depression increased the risk of anxiety symptoms, as did a prior history of anxiety disorders. Women who showed symptoms of anxiety expressed a greater fear of childbirth than those who showed no such symptoms. The adverse effect of anxiety on the fetus and the pregnant woman is not fully understood. In a review, Alder and colleagues examined 35 studies published between 1990 and 2005 and found that enhanced levels of anxiety symptoms during pregnancy contributed independently of other biomedical risk factors to adverse obstetric, fetal, and neonatal outcomes [10].

Findings from animal studies have shown a link between antenatal stress, measured as the occurrence of major life events, and impact on behavior and emotional adjustment as well as cognitive impairment of the offspring both in childhood but also later in adulthood [11]. The interpretation of these findings is that changes in the function of the hypothalamic – pituitary - adrenal (HPA) axis account for these relationships [12].

In a review by Monk et al., evidence for the impact of human maternal distress on fetal and infant outcomes through epigenetic mechanisms was outlined. Prenatal exposure to maternal anxiety and depression can have lasting effects on infant development with consequences for risk of psychopathology [13]. The recurrence rate and chronicity of anxiety conditions are high. In addition, many women with anxiety are not clinically recognized which means that many children are exposed to maternal anxiety not just temporarily but during a substantial part of their childhood.

Hence, the findings from both animal and human studies indicate that anxiety have a negative influence on both child and mother. In a long-term perspective these findings provide a solid foundation on which to form hypotheses concerning how anxiety may shape risks for coming generations. To our knowledge there are no studies based on a national population investigating whether there is an impact beyond the two generations i.e. mother and child.

The aim of the present study was therefore to investigate the intergenerational transmission of anxiety in a national population comprising three generations. More specifically, the primary aim was to investigate the risk for being diagnosed with anxiety given that previous generation(s) of mothers had been diagnosed with anxiety. Additional aim was to examine the use of specialized health care among third generation children, if either or both previous generation of mothers had been diagnosed with anxiety.

## Methods

### Data collection

The information on all the participants in this study was retrieved from Swedish population-based registries. All Swedish residents are given unique personal identification numbers that allow us to individually link the information about each person from different registers.

Background variables such as educational level, marital status, and parity were registered in the Swedish Medical Birth Register (MBR) at the time of admission to antenatal care. From the other registers we collected information on parents’ country of birth, the women’s marital status, highest attained educational level as well as anxiety diagnosis and use of specialized health care. More specifically, data were collected from the following registers:

- The Swedish Medical Birth Register (MBR): Medical information on all births since 1973 and onwards has been stored in the MBR which is held by the Swedish National Board of Health and Welfare [14].
- The Total Population Register (TPR): The TPR is held by Statistics Sweden and was established in 1968 [15, 16]. The register contains information on variables such as births, deaths, migrations, and marital status.
- The Causes of Death Register: The Causes of Death Register, which is held by the Swedish National Board of Health and Welfare, contains information on the cause of death and was established in 1961 [17, 18].
- The Education Register and the Population and Housing Census: Since 1985, Statistics Sweden has continuously collected information on the educational level of the population in the Education Register [19–21].
- The National Patient Register (NPR): The NPR was originally established in 1964, with its main focus being on psychiatric diagnoses. From 1987 all inpatient visits are included and in 2001 outpatient visits were added to the register.

These registers have all been evaluated [14, 22–26]; The Inpatient Register was most recently evaluated by Ludvigsson et al. in 2011, who concluded that the register is of good quality with a high validation rate [22]. Similarly, The Education Register, The Medical Birth Register, The Total Population Register and The Cause of Death Register have been evaluated and deemed to be of a high quality.

### Study population

For the purpose of this study we selected all women who gave birth between 1973 and 1977 (*n* = 169,782, all of these women were born between 1924 and 1963), their daughters (*n* = 244,152 who were born between 1973 and 1977), and subsequently also the offspring of the daughters (*n* = 381,953 who were born between 1987 and 2012); all three groups were followed until the 31 December, 2012. At time of data collection no further data were available by the registers holder for a longer follow-up, since data have to be validated before being released to researchers. The data collection included identifying if the two first generations of women had become mothers, and their socio-demographic characteristics, whether the individuals in the study (all generations) had been diagnosed with an anxiety diagnosis during the entire follow-up time, as well as how many times they had used specialized hospital care, either as an outpatient or inpatient resulting in an anxiety diagnosis.

### Diagnoses

To study these women and third-generation children, we used The National Patient Register (NPR), which contains all psychiatric inpatient care diagnoses and from 2001 all outpatient-diagnoses in a hospital setting.

The anxiety diagnoses are based on the Swedish version of *The International Classification of Diseases* (ICD) from the World Health Organization [27]. Between 1969 and 1986 ICD version 8 was in use. In 1987 a new version of ICD was released and was in use until 1997 when the health care system changed the ICD-version used from ICD-9 to ICD-10. During this year ICD-9 and ICD-10 were used interchangeably. Anxiety diagnoses in ICD-8 were identified as codes 300–301, 305–308, ICD-9 codes were identified as codes 290–319 and in ICD-10 codes F40-F42 (24), this includes diagnoses such as phobic anxiety, panic disorders, generalized anxiety disorder, and obsessive compulsive disorders. Therefore we searched for both ICD-9 and 10 codes in 1997 and only ICD-10 in 1998 to 2004. ICD-8 and ICD-9 codes were translated to ICD-10 using a conversion table [27].

### Definitions

Anxiety disorders in all three generations were divided into two categories according to ICD-10 (24), diagnosis present and diagnosis not present. Socio-demographic variables on the grandmothers included parity (previous children/no previous children), highest attained level of education (Elementary, High school and Graduate/Post-graduate), region of origin (Nordic/Non-Nordic), marital status (Married, Unmarried and Divorced/widowed), age when giving birth (< 20 years, 20–26 years, 27–33 years and > 33 years). The same set of socio-demographic factors was collected for the mothers, except for origin since all mothers were born in Sweden.

In addition to anxiety diagnosis according to ICD-10, patient data regarding the total number of outpatient and inpatient visits were collected for the third-generation children, and arbitrarily cut off incidences of 0–1 visits/≥ 2 visits for inpatient data and 0–10 visits/≥11 visits for out-patient data were chosen. These served as proxies for overall morbidity among the third-generation children.

### Statistical analysis

To examine the risk for anxiety disorder in the studied generation of women and their children we analyzed the data by using Pearson’s chi-square to analyze bivariate differences. Data were also analyzed by unadjusted as well as adjusted logistic regression in order to estimate the odds ratio of being diagnosed with anxiety, each generation modeled separately. The dependent variable was the presence of anxiety diagnoses and the independent variables were educational level, marital status, and parity. For the third-generation children the same set of independent variables were used to estimate the odds ratios for having a larger amount of inpatient or outpatient visits to the hospital, or being diagnosed with anxiety. These models also included an additional independent variable; a three level indicator on the presence of anxiety in the two previous generations (only 1st generation woman diagnosed with anxiety, only 2nd generation woman diagnosed with anxiety, or both generations diagnosed with anxiety).

All analyses were performed using SPSS, version 22.0 (IBM SPSS Inc., Armonk, NY).

## Results

The study population is shown in Table 1. Approximately 4% of the second-generation mothers and 6% of the first-generation mothers had, at some point, been diagnosed with anxiety. Out of 381,953 children a total of 65,838 of the children (i.e. the third-generation) had had more than 10 outpatient visits for at some kind of medical or psychiatric disorder (median = 5, range = 1–261) and 53,649 had had two or more inpatient visits for some kind of disorder (median = 10, range = 2–504) as presented in Table 1.

**Table 1:** The study population encompassing two generations of women and their offspring

	Study population
	n (%)
Total no. of children (third generation)	381,953
No. of children with anxiety diagnosis	749 (0.2)
No. of children with ≥10 outpatient visits	65,838 (17.2)
No. of children with ≥2 inpatient visits	53,649 (14.0)
Total no of mothers (second generation)	244,153
No of mothers with anxiety diagnosis	10,285 (4.2)
Total no. of grandmothers (first generation)	169,782
No of grandmothers with anxiety diagnosis	10,301 (6.1)

The background characteristics of the first- and second-generation mothers are shown in Table 2.

**Table 2:** Socio-demographic characteristics of the study participants – limited to study persons born between 1973 and 1977, indifferent on future child

	Anxiety	Anxiety		*p*-value^a^
		No	Yes	
		n (%)	n (%)	
First-generation mothers				
Educational level	Elementary	50,015 (22.1)	2539 (25.2)	< 0.001
	High school	110,512 (48.9)	4937 (49.1)	
	Graduate/post-graduate	65,699 (29.0)	2589 (25.7	
Civil status	Married	190,869 (84.2)	7830 (78.6)	< 0.001
	Unmarried^b^	17,493 (7.7)	990 (9.9)	
	Divorced/widowed	18,261 (8.1)	1144 (11.5)	
Parity	No previous children	102,328 (43.8)	4444(43.2)	0.275
	Previous children	131,540 (56.2)	5841 (56.8)	
Age when giving birth	< 20	16,099 (6.9)	1018 (9.9)	< 0.001
	20–26	111,212 (47.6)	4792 (46.6)	
	27–33	88,450 (37.8)	3626 (35.3)	
	> 33	18,107 (7.7)	849 (8.3)	
Origin	Nordic	9905 (4.2)	408 (4.0)	0.185
	Non-Nordic	223,963 (95.8)	9877 (96.0)	
Second-generation women				
Educational level	Elementary	9954 (4.4)	1513 (15.0)	< 0.001
	High school	89,281 (39.2)	4466 (44.2)	
	Graduate/post-graduate	128,453 (56.4)	4129 (40.8)	
Civil status	Married	123,020 (52.6)	4596 (44.7)	< 0.001
	Unmarried^a^	107,549 (46.0)	5387 (52.4)	
	Divorced/widowed	3298 (1.4)	302 (2.9)	
Parity	No previous children	94,215 (54.9)	3891 (56.8)	0.003
	Previous children	77,348 (45.1)	2964 (43.2)	
Age when giving birth	< 20	3220 (1.9)	422 (6.2)	< 0.001
	20–26	37,956 (22.1)	2119 (30.9)	
	27–33	95,051 (55.4)	3093 (45.1)	
	> 33	35,336 (20.6)	1221 (17.8)	
Childbirth	No	48,825 (20.9)	2893 (28.1)	< 0.001
	Yes	185,043 (79.1)	7392 (71.9)	
First-generation mother diagnosed with anxiety	Yes	13,480 (5.8)	8884 (86.4)	< 0.001
	No	220,388 (94.2)	1401 (13.6)	
First-generation mother, no. of visits to hospital due to anxiety	0–3 visits	104,551 (44.7)	3162 (30.7)	< 0.001
	4- visits	129,317 (55.3)	7123 (69.3)	< 0.001
Second-generation women, no. of visits to hospital due to anxiety	0–3 visits	133,878 (57.2)	652 (6.3)	
	4- visits	99,990 (42.8)	9633 (93.7)	

^a^Chi2-test

^b^The category Unmarried includes women cohabiting with the child’s father though they are not legally married and women who are considered single (i.e. not married or cohabiting with the child’s father)

We found that second-generation women diagnosed with anxiety were more likely to have had mothers with a lower level of education, with higher rates of divorce or widowhood, and higher rates of having been diagnosed with anxiety than the mothers of second-generation women who had not been diagnosed with anxiety.

Moreover, second-generation women diagnosed with anxiety were less likely to have given birth during the study period, and those who had given birth to at least one child, had reproduced at a younger age than women who had not been diagnosed with anxiety. Limiting the study population to second-generation women who had had at least one child it was found that second-generation mothers diagnosed with anxiety were more likely to have had mothers with a lower level of education, more often had mothers who had been divorced or become a widow, and who had had their child at a younger age, Table 3. They themselves also had a lower level of education, were more often divorced or widowed, and had had children at a younger age compared to mothers not diagnosed with anxiety. Furthermore, second-generation mothers diagnosed with anxiety were also more prone to have children who had been diagnosed with anxiety and children who more frequently received specialized medical care; both as inpatients and outpatients.

**Table 3:** Socio-demographic characteristics of the study participants, limited to women born between 1973 and 1977 who had become mothers

	Anxiety	Anxiety		*p*-value
		No	Yes	
		n (%)	n (%)	
First-generation mothers				
Educational level	Elementary	87,638 (22.6)	4197 (26.7)	< 0.001
	High school	191,237 (49.3)	7869 (50.0)	
	Graduate/post-graduate	108,767 (28.1)	3661 (23.3)	
Civil status	Married	322,053 (84.8)	12,314 (79.0)	< 0.001
	Unmarried^a^	27,341 (7.2)	1458 (9.4)	
	Divorced/widowed	30,443 (8.0)	1811 (11.6)	
Parity	No previous children	107,573 (43.5)	7009 (43.6)	0.720
	Previous children	221,502 (56.5)	9049 (56.4)	
Age when giving birth	< 20	30,254 (7.7)	2020 (12.6)	< 0.001
	20–26	191,791 (48.9)	7857 (48.9)	
	27–33	142,058 (36.2)	5013 (31.2)	
	> 33	27,972 (7.1)	1168 (7.3)	
Origin	Nordic	379,255 (96.7)	15,459 (96.3)	0.001
	Non-Nordic	12,820 (3.3)	599 (3.7)	
Second-generation mothers				
Educational level	Elementary	17,193 (4.4)	2452 (15.3)	< 0.001
	High school	156,213 (39.9)	7297 (45.5)	
	Graduate/post-graduate	218,399 (55.7)	6297 (39.2)	
Civil status	Married	254,795 (65.0)	9559 (59.5)	< 0.001
	Unmarried^a^	129,781 (33.1)	5766 (35.9)	
	Divorced/widowed	7498 (1.9)	733 (4.6)	
Parity	No previous children	174,958 (47.7)	6979 (46.4)	0.002
	Previous children	191,962 (52.3)	8054(53.6)	
Age when giving birth	< 20	8067 (2.2)	1003 (6.7)	< 0.001
	20–26	86,576 (23.6)	4862 (32.3)	
	27–33	203,016 (55.3)	6706 (44.6)	
	> 33	69,261 (18.9)	2461 (16.4)	
Second-generation mother diagnosed with anxiety	Yes	23,073 (5.9)	2172 (13.5)	< 0.001
	No	369,002 (94.1)	13,886 (86.5)	
First-generation mother, no. of visits to hospital due to anxiety	0–3 visits	169,608 (43.3)	4721 (29.4)	< 0.001
	4- visits	222,467 (56.7)	11,337 (70.6)	
Second-generation mother, no. of visits to hospital due to anxiety	0–3 visits	171,508 (43.7)	346 (2.2)	< 0.001
	4- visits	220,567 (56.3)	15,712 (97.8)	
Children (3rd generation)				
Gender	Boy	188,199 (51.3)	7753 (51.6)	0.498
Outpatient visits	Girl	178,721 (48.7)	7280 (48.2)	
	0–10	305,181 (83.2)	10,934 (72.7)	< 0.001
	≥11	61,739 (16.8)	4099 (27.3)	
Inpatient visits	0–1	316,924 (86.4)	11,380 (75.7)	< 0.001
	≥2	49,996 (13.6)	3653 (24.3)	
Anxiety diagnosis (child)	Yes	391,447 (99.8)	15,397 (99.2)	< 0.001
	No	628 (0.2)	121 (0.8)	

^a^The category Unmarried includes women cohabiting with the child’s father though they are not legally married and women who are considered single (i.e. not married or cohabiting with the child’s father)

The unadjusted odds ratio of having been diagnosed with anxiety was more than twice as high among second-generation mothers who themselves had mothers who at some point had been diagnosed with anxiety (OR = 2.58, 95% CI = 2.43–2.74), Table 4. Among the 3rd generation children, the lowest increased odds ratios were seen for those for whom only the first-generation mothers had been diagnosed with anxiety (OR = 2.14 95% CI 1.68–2.73), Table 4, while children where both 1st and 2nd generation women had been diagnosed with anxiety exhibited the highest odds ratio for being diagnosed with anxiety (OR = 7.74, 95% CI = 5.18–11.59).

**Table 4:** Unadjusted odds ratios (OR) and corresponding 95% confidence intervals (CI) on the intergenerational effect of anxiety disorder in three generations^a,b,c^

	Total	Boys	Girls
	OR (95% CI)	OR (95% CI)	OR (95% CI)
OR Second-generation mother diagnosed with anxiety			
First-generation mother diagnosed with anxiety	2.58 (2.43–2.74)	2.61 (2.36–2.88)	2.56 (2.31–2.84)
First-generation mother not diagnosed with anxiety	Reference	Reference	Reference
OR child (3rd generation) has inpatient care^d^			
First- and second-generation mother diagnosed with anxiety	2.64 (2.40–2.91)	2.36 (2.06–2.69)	3.02 (2.63–3.47)
Only second-generation mother diagnosed with anxiety	1.98 (1.90–2.07)	1.92 (1.81–2.03)	2.08 (1.95–2.12)
Only first-generation mother diagnosed with anxiety	1.30 (1.25–1.35)	1.25 (1.19–1.32)	1.36 (1.29–1.44)
None diagnosed with anxiety	Reference	Reference	Reference
OR child (3rd generation) has outpatient care^e^			
First- and second-generation mother diagnosed with anxiety	2.21 (2.01–2.43)	2.28 (2.01–2.59)	2.14 (1.86–2.46)
Only second-generation mother diagnosed with anxiety	1.83 (1.76–1.90)	1.79 (1.70–1.89)	1.87 (1.77–1.99)
Only first-generation mother diagnosed with anxiety	1.23 (1.19–1.27)	1.22 (1.16–1.28)	1.24 (1.81–1.31)
None diagnosed with anxiety	Reference	Reference	Reference
OR child (3rd generation) diagnosed with anxiety			
First- and second-generation mother diagnosed with anxiety	7.74 (5.18–11.59)	6.73 (3.15–14.35)	8.40 (5.21–13.53)
Only second-generation mother diagnosed with anxiety	4.63 (3.72–5.75)	4.40 (2.97–6.52)	4.92 (3.78–6.40)
Only first-generation mother diagnosed with anxiety	2.14 (1.68–2.73)	2.36 (1.57–3.57)	2.06 (1.52–2.80)
None diagnosed with anxiety	Reference	Reference	Reference

^a^All second-generation mothers are born between 1973 and 1977

^b^All children (third-generation) are born between 1987 and 2012

^c^All first-generation mothers are born between 1924 and 1963

^d^Inpatient care categorized into 0–1 visits and ≥ 2 visits

^e^Outpatient care categorized into 0–10 visits and ≥ 11 visits

Among the third generation children, the odds ratios of being diagnosed with anxiety, having more than two inpatient visits and/or having more than 10 outpatient visits were highest among those children for whom both the first- and second-generation mothers had been diagnosed with anxiety (OR = 2.64, 95% CI = 2.40–2.91 and OR = 2.21, 95% CI = 2.01–2.43, respectively) compared to children where none of the previous generations had an anxiety diagnosis, Table 4. Stratifying by gender revealed that the increased likelihood of having an anxiety diagnosis, having more than two inpatient visits and/or more than 10 inpatient visits were approximately the same among both boys and girls whose mothers and grandmothers had been diagnosed with anxiety, in comparison to boys and girls where none of the previous generations had had an anxiety diagnosis, Table 4.

After adjusting for socio-demographic factors (educational level, marital status and parity) the odds ratios decreased. However, having a mother and/or a grandmother diagnosed with anxiety still remained an important factor in determining whether the child had a relatively higher number of visits for specialized medical care and an anxiety diagnosis, Table 5. If both mother and grandmother were diagnosed with anxiety the odds ratio was almost twice as high for in- and out-patient care (OR = 1.94, 95% CI = 1.75–2.14 and OR = 1.74, 95% CI = 1.58–1.92, respectively) while having been diagnosed with anxiety the odds ratio was threefold (OR = 3.11, 95% CI = 2.04–4.75) compared to children where none of the previous generations had been diagnosed with anxiety, Table 5. Moreover, in the gender stratified analysis, both boys and girls where the two previous generations had been diagnosed with anxiety, had an increased likelihood of being diagnosed with anxiety, having two or more inpatient visits and/or 10 or more outpatient visits compared to children where none of the previous generations had been diagnosed with anxiety. This increased likelihood was approximately of the same magnitude among both boys and girls, Table 5. To further elucidate the impact of previous generation’s anxiety diagnosis, the 3rd generation was divided into two strata, 0–12 year olds, and 13 or older. This analysis validates the increased risk for an anxiety diagnosis among the third generation children if any or both of the previous generations have been diagnosed with anxiety, Table 6. This was especially evident among girls, where the ORs, were higher and with a narrower confidence interval, indicating a more reliable estimate. Also, among both boys and girls, there were increased risks for having been diagnosed with anxiety in the younger groups (0–12 years of age), but the estimates did not always reach statistical significance or had very wide confidence intervals. This was probably due to the limited number of children having received specialized hospital care due to anxiety.

**Table 5:** Adjusted odds ratios (OR) and corresponding 95% confidence intervals (CI) on the intergenerational effect of anxiety disorder in three generations^a,b,c^

	Total	Boys	Girls
	OR (95% CI)	OR (95% CI)	OR (95% CI)
OR second generation mother diagnoses with anxiety			
First-generation mother diagnosed with anxiety	2.20 (2.04–2.38)	2.20 (1.98–2.44)	2.22 (1.99–2.47)
First-generation mother not diagnosed with a anxiety	Reference	Reference	Reference
OR child (3rd generation) has inpatient care^d^			
First- and second-generation mother diagnosed with anxiety	1.94 (1.75–2.14)	1.76 (1.53–2.02)	2.17 (1.88–2.52)
Only second-generation mother diagnosed with anxiety	1.66 (1.59–1.74)	1.63 (1.53–1.73)	1.72 (1.60–1.83)
Only first-generation mother diagnosed with anxiety	1.14 (1.10–1.19)	1.11 (1.05–1.07)	1.19 (1.12–1.26)
None diagnosed with anxiety	Reference	Reference	Reference
OR child (3rd generation) has outpatient care^e^			
First- and second-generation mother diagnosed with anxiety	1.74 (1.58–1.92)	1.84 (1.61–2.10)	1.63 (1.41–1.89)
Only second-generation mother diagnosed with anxiety	1.62 (1.55–1.69)	1.62 (1.52–1.71)	1.63 (1.53–1.74)
Only first-generation mother diagnosed with anxiety	1.11 (1.07–1.15)	1.12 (1.06–1.18)	1.11 (1.05–1.17)
None diagnosed with anxiety	Reference	Reference	Reference
OR child (3rd generation) diagnosed with anxiety			
First- and second-generation mother diagnosed with anxiety	3.11 (2.04–4.75)	2.97 (1.37–6.45)	3.14 (1.88–5.22)
Only second-generation mother diagnosed with anxiety	2.54 (2.01–3.20)	2.38 (1.57–3.62)	2.63 (1.99–3.49)
Only first-generation mother diagnosed with anxiety	1.46 (1.14–1.88)	1.57 (1.02–2.42)	1.42 (1.04–1.93)
None diagnosed with anxiety	Reference	Reference	Reference

^a^All second-generation mothers are born between 1973 and 1977

^b^All children (third-generation) are born between 1987 and 2012

^c^All first-generation mothers are born between 1924 and 1963

^d^Inpatient care categorized into 0–1 visits and ≥ 2 visits

^e^Outpatient care categorized into 0–10 visits and ≥ 11 visits

**Table 6:** Unadjusted odds ratios (OR) and corresponding 95% confidence intervals (CI) on the intergenerational effect of anxiety in three generations, stratified by age and gender of the child (3rd generation) ^a,b,c^

	Total		Boys		Girls	
	0–12 years	13- years	0–12 years	13- years	0–12 years	13- years
OR child (3rd generation) diagnosed with anxiety						
First- and second-generation diagnosed with anxiety	6.41(2.03–20.23)	3.74 (2.42–5.77)	3.83 (0.53–27.23)	9.07 (2.35–40.14)	3.58 (1.57–8.19)	3.96 (2.36–6.62)
Second-generation mother diagnosed with anxiety	2.13 (0.99–4.58)	3.14 (2.49–3.96)	1.06 (0.26–4.35)	3.54 (1.40–8.95)	3.48 (2.28–5.30)	3.06 (2.32–4.05)
Only first-generation mother diagnosed with anxiety	2.07 (1.14–3.77)	1.51 (1.16–1.98)	2.73 (1.35–5.52)	1.20 (0.37–3.87)	1.55 (0.93–2.58)	1.51 (1.10–2.07)
None diagnosed with anxiety	Reference	Reference	Reference	Reference	Reference	Reference

^a^All second-generation mothers are born between 1973 and 1977

^b^All children (third-generation) are born between 1987 and 2012

^c^All first-generation mothers are born between 1924 and 1963

## Discussion

In this nationwide population-based study we have been able to shed light on the intergenerational transmission of anxiety in three generations. It was found that the transmission of anxiety from one generation to the next is very high. More specifically, we found that women (2nd generation) who had mothers (1st generation) with an anxiety disorder were more than twice as likely to have an anxiety disorder themselves in comparison to all other women. Moreover, in the third generation, among children born to mothers with an anxiety disorder, the unadjusted odds ratio of being diagnosed with anxiety was more than four times higher compared to children (3rd generation) where none of the previous generations had been diagnosed with anxiety disorders. If both the mother and the grandmother had had an anxiety disorder the unadjusted odds ratio for the child having a diagnosis of anxiety close to eight times higher. Adjusting confounding factors such as marital status, educational level and parity, the odds ratios decreased but still remained elevated at three, and two and a half times higher, respectively, compared to children where none of the previous generations had an anxiety diagnosis. An explanation for this might be that the specialist care is more prone to investigate a child’s problem and to be more precise in collecting medical history in order to diagnose appropriately. Another possible explanation might be that the parents who seek help for anxiety related disorders are able to present a child’s problem or help the child to present his or her problems in an accurate way since they themselves have knowledge of anxiety disorders and how anxiety has affected them. Lastly, the child might also be able to present his or hers problems more systematically when seeking help.

The children in the third generation of mothers with anxiety also had a higher consumption of relatively advanced medical care such as inpatient, and outpatient specialist care. With the exception of anxiety disorders, the children’s exact medical diagnoses and interventions are outside the scope of this study and were therefore not investigated. However, a study of the full medical record might be expected to show a higher level of somatic ill-health that in turn might be a signal of additional problems within the family, the personality of the child, and even childhood experiences outside the family [28].

The transmission of anxiety from parents to offspring is well known, but the underlying processes are poorly understood [29–32]. Most studies have evaluated the risks from a two-generation perspective and there is no evidence that allows us to determine the relative importance of genetic factors and environmental factors, respectively, on the transmission of anxiety disorders for the 3rd generation. In this study we only have information on anxiety disorders in the maternal probands [13, 29–32]. Little is known about the effect of paternal anxiety disorders on children’s psychopathology but in a study by Cooper et al. 2006 it was evident that there is a strong familiality of anxiety disorders in general but the impact of maternal anxiety was more evident [30]. In a Swedish twin study, the authors argue that environmental transmission from parents is stronger than genetic factors because the children learn an anxious behavior from their parents through modeling [33]. Different paths of environmental transmission are plausible. For example, maternal anxiety has been shown to be associated with reduced tolerance to negative emotions in children [34]. Anxious mothers were also shown to have lower expectations of their children’s performance compared to non-anxious mothers [34]. Another study found anxiety in children to be associated with overinvolved and critical parenting [35]. Whether these parenting behaviors are influenced by anxiety in the offspring, or whether the rearing environment increases the risk of the development of anxiety in children is not fully understood. While parenting styles were not investigated in this study, family structure and socioeconomic factors such as education was found to impact the risk for anxiety. These findings underline the multifactorial etiology of anxiety conditions, and the importance to address the psychosocial environment. Moreover, there are studies suggesting that different genetic factors impact the development of anxiety disorders during childhood, adolescence and early adulthood respectively [36], indicating genetic innovation and attenuation or possibly epigenetic mechanisms. Kendler et al. (2008) found support for a developmentally dynamic hypothesis of genetic effects on anxiety and depression, which could explain the low level of homotypic continuity of anxiety disorders from childhood to adulthood [36]. In a recent study by Weissman and colleagues [37], it was found that offspring to depressed parents had a risk for major depression and that the period of peak for first onset was between ages 15–25. Onsets before adolescence were uncommon; there was also an increase in morbidity and mortality indicating a greater vulnerability in general [37].

In a prevalence study on mental health, the results showed that among individuals with depression or anxiety, around one third did not receive treatment. Comorbidity was associated with higher symptom severity and lower health-related quality of life and that these mental disorders form a unit and thus depression and anxiety can be seen together as a rule rather than an exception. [4] Overall, comorbidity is of great importance to acknowledge and to investigate. Mainly in order to understand the effect of comorbidity, but also to better screen the patient and design individual treatment and care for the patients. A high recurrence rate of anxiety conditions has also been found [38].

Reproductive events and particularly childbirth are risk factors for acquiring mental disorders for women. In a register study from Denmark it was concluded that primiparous women had an increased risk of incident-related hospital admission to a psychiatric hospital for a mental disorder through the first 3 months after childbirth but among fathers there was no increase of severe mental disorders that required their admission to a hospital [39].

Animal as well as human studies have shown associations between antenatal stress and or anxiety development and behavioral/emotional disturbance in the child. Yet, the strength of this link was unclear since these studies did not examine the covariance of antenatal risks and did not distinguish between ante- and postnatal stress. The large Avon Longitudinal Study of Parents and Children (ALSPAC) cohort showed a strong relationship between maternal anxiety in late pregnancy and behavioral/emotional problems in their children at age four [40].

Animal studies have shown that offspring of mothers who have suffered antenatal stress are over-reactive to stressors and hypersecrete cortisol compared with controls. Both the behavioral and physiological disturbances last into adulthood in rodents and for several years, suggesting that the HPA axis can be ‘programmed’ during the fetal period [12].

In most prevalence studies there are, generally, significant gender differences with mental health disorders being more common in women than in men. The effect on the families and generations might therefore be of significance as our results show. Since mental health problems seem to have an impact on subsequent generations it is of the utmost importance to detect and treat anxiety.

A limitation of this study is that only diagnoses set at hospitals or specialist clinics such as psychiatric outpatient clinics are used. Therefore one can suspect that the true percentage of individuals with an anxiety disorder is higher as a number of individuals may have been diagnosed and treated by their general practitioners. Thus, only including diagnoses from a hospital or specialist clinic setting implies that only the most severe forms of anxiety are included. This may cause an overestimation of the intergenerational transmission. Also, misclassification problems caused by unrecorded cases and/or incorrect registration of diagnostic codes are known limitations in register studies. If so, the incorrect registration is random and not systematic. Moreover, the children (third-generation) in the present study were born between 1987 and 2012. While anxiety disorders develop relatively early in life with a mean onset age of 11 years [1], the results are strengthened by the presence of an intergenerational effect already evident at an early age. Another limitation is the lack of information of the timing of anxiety in relation to childbirth. Anxiety symptoms during pregnancy have been shown to increase the risk for adverse obstetric, fetal, and neonatal outcomes [10], while maternal anxiety during early childhood might impact the children through negative or over-controlling parenting behaviors [35]. Moreover, recurrent or chronic anxiety episodes would have a greater impact on both the woman and her children, compared to a single anxiety episode.

## Conclusion

Second-generation mothers diagnosed with anxiety had children who had been diagnosed with anxiety and children who more frequently received medical care; both as inpatients and outpatients. Anxiety disorders have a three-generational effect. Since intergenerational effect on anxiety is high and in order to minimize the risk for further transmission of anxiety disorders, increased awareness and generous use of effective treatment regimes might be important.
